# Improved Multi-GNSS PPP Software for Upgrading the DEMETRA Project Time Monitoring Service

**DOI:** 10.3390/s19204389

**Published:** 2019-10-11

**Authors:** Wei Huang, Pascale Defraigne, Giovanna Signorile, Ilaria Sesia

**Affiliations:** 1 Politecnico di Torino, 10129 Torino, Italy; 2 Observatoire Royal de Belgique, 1180 Brussels, Belgium; pascale.defraigne@oma.be; 3 Istituto Nazionale di Ricerca Metrologica, 10135 Torino, Italy; g.signorile@inrim.it (G.S.); i.sesia@inrim.it (I.S.)

**Keywords:** precise point positioning, GNSS, time transfer, frequency transfer, Galileo, constrained PPP, integer PPP, DEMETRA, time service

## Abstract

The H2020 DEMETRA project provides short latency clock monitoring services to the time users using the Atomium precise point positioning (PPP) software developed by the Royal Observatory of Belgium. In this paper, three recent updates of the current Atomium software are introduced: adding Galileo signals in the PPP computation; the option to constrain the receiver clock; PPP with integer ambiguity resolution. The advantages of these updates are demonstrated: Combining the Galileo and global positioning system (GPS) signals for PPP time transfer will further improve the frequency stability inside the computation batch; PPP with receiver clock constraint is not only used to reduce the short-term noise of the clock measurements but can also be used for some specific applications to a keep continuous clock solution in the computation batch or retrieve correct clock measurements from extremely noisy environments; the integer PPP allows a continuous clock solution, and improves the mid-term and long-term stability of the frequency transfer compared to the current PPP frequency transfer techniques.

## 1. Introduction

The global navigation satellite systems (GNSS) include the United States of America’s global positioning system (GPS), Russian’s global navigation satellite system (GLONASS), Europe’s Galileo and China’s BeiDou navigation satellite system. They are well known for their positioning services with global coverage. With the GNSS precise point positioning (PPP) techniques, the positioning accuracy can reach centimeter-level accuracy [1]. Another important function that GNSS provides to the user is the timing service, based on the accurate and reliable GNSS time references, e.g., GPS system time (GPST), and their predicted difference to the Coordinated Universal Time (UTC) [2].

GNSS are widely used for accurate time and frequency dissemination, synchronization, and remote clock comparison (called time transfer in time metrology). In the industrial domain, GNSS time synchronization is widely used in the telecom network, energy grid, and financial system [3] and has proved its advantages in various specific applications, e.g., time synchronization of the intelligent transportation systems [4] and spacecraft [5]. In the scientific domain, clock comparison with GNSS is essential for the computation of International Atomic Time (TAI), with more than 50% of time laboratories contributing to TAI and UTC equipped by GNSS receivers for their time link comparisons [6]. GNSS timing is also the primary choice for some scientific activities in which all the stations from a given network observing the same event need to be highly time-synchronized, e.g., seismic monitoring [7], deep space tracking [8], and neutrinos speed measurement [9].

Many users rely on GNSS code measurements for the time synchronization purpose, this method has a statistical uncertainty of several nanoseconds over one day [10]. Better performance of GNSS time transfer is achieved by PPP techniques, which use both dual-frequency carrier phase and code measurements from the GNSS signals and the more precise satellite products instead of the broadcast navigation message for the analysis. The statistical uncertainty of this method can reach 100 ps over one day [6,11].

DEMETRA (*DEM*onstrator of *E*GNSS services based on *T*ime *R*eference *A*rchitecture) [12] is a project funded by the European Union in the Horizon 2020 program coordinated by the Italian National Metrology Institute (INRIM) that involved 16 European partners, including the Royal Observatory of Belgium (ORB). The DEMETRA project aims at providing the end-users in both industrial and scientific domains with improved and new time services, some of them being based on the European GNSS. Nine different timing services with improved accuracy or availability and any requisite as certification or redundancy were designed, developed, and tested. Most of the time, services are currently still operative through their infrastructures maintained at the INRIM premises. As an example, INRIM implemented the fiber link between the financial district in Milan and INRIM for UTC time distribution service [13].

One particular service in DEMETRA called “Time Monitoring and Steering”, provides the user, in quasi-real-time, the time difference between the user atomic clock and the reference time scale of DEMETRA from an hourly PPP solution. This PPP solution is based on the data from the user geodetic GNSS receiver transmitted hourly to the DEMETRA platform. With the data provided by the service, the user can make sure its clock is highly synchronized to the DEMETRA reference and continue being alerted to any possible non stationarities on its clock, namely phase or frequency jumps. Meanwhile, the service offers the necessary quality monitoring data (such as satellite sky plot, multipath, cycle slips, etc.) visible directly on the personal area of the DEMETRA web page. An example of part of the user personal page is attached in the Appendix A, and all the details can be found on the homepage of the DEMETRA project [14]. In addition, some parameters are sent to the users to steer their clock to UTC if needed. Because these steering parameters are computed based on the monitoring data, in this paper, we focus on the algorithms of the time monitoring part of the service. 

The core software in the DEMETRA timing monitoring service is called Atomium, which is developed by ORB [11]. Atomium is a GPS+GLONASS PPP software dedicated to GNSS time transfer and geodetic positioning. It uses the least-square method to estimate the following parameters: receiver clock error at each epoch; user antenna position for the whole daily data batch; tropospheric zenith wet delay at defined interval, and float phase ambiguities. The software uses satellite products made available from International GNSS service (IGS) and estimates the clock errors as “IGS time-user clock”. On the DEMETRA side, the computation “IGS time-DEMETRA reference” is also carried out with PPP, for the same time period. Differentiating these two quantities, we obtain estimates of the user clock with respect to the DEMETRA reference. The user clock can be either the internal clock of the GNSS receiver or an external clock (e.g., hydrogen maser, cesium, rubidium atomic clock) used to drive the GNSS receiver at the user side. For convenience, all these clocks are called user clock or receiver clock, in general, in this paper. The DEMETRA reference time is chosen to be the UTC(IT) time scale that feeds the GNSS receiver at the INRIM side. UTC(IT) is the local realization of UTC made by the INRIM and is based on a hydrogen maser steered on UTC.

Atomium PPP has been dedicated to precise time and frequency transfer for more than 10 years [15]. Currently, the PPP time transfer is mainly limited by the time discontinuity at the daily batch boundary, which is caused by the averaging of the noisy code measurements in the daily batches [16]. A common way to avoid the daily boundary discontinuities is to extend the estimation batch to multi-day (e.g., with PPP software developed by Natural Resources Canada (NRCAN) [17]). However, the boundaries still exist at the end of the multi-day batches. In addition, this method may cause the long-term artificial variation of the clock estimation, introducing additional random walk noise inside the batch [18]. Moreover, while being the state-of-the-art GNSS technique for modern high-quality clock comparison, short-term stability of the PPP is currently limited by the thermal noise in the carrier phase data. Further developments are, therefore, still needed to improve the stability of the PPP clock solution and mitigate the boundary issue. In this paper we present three updated versions of the Atomium PPP software that we have integrated into DEMETRA for improving the time monitoring part of the service: (i) Atomium Galileo PPP; (ii) Atomium constrained PPP; (iii) Atomium integer PPP. The basic ideas of the proposed new algorithms have been presented in [19], while they have not been included in any proceedings paper. In the current work, the mathematic principles of these improved PPP algorithms are described in detail, and results based on experimental data are presented enabling the evaluation of the improvements of the new methods and to define possible application scenarios.

Atomium Galileo PPP includes Galileo measurements in the PPP computation, in addition to the GPS and GLONASS ones in the old version. Though the Galileo satellite system is still not fully operational for the moment, comparable performance to GPS in time transfer with code-only measurements has already been demonstrated in [20]. This paper presents the impact of using GPS+Galileo data in Atomium PPP for time and frequency transfer.

Second, Atomium constrained PPP will be presented. This approach further constrains the receiver clock with the estimated frequency offset and drift, to retrieve the short-term stability of the measurements on the clocks with good quality (e.g., hydrogen maser), by restricting the impact of thermal noise on the receiver clock solutions [21]. Applying a stochastic model in kinematic PPP has shown improvement in the kinematic positioning [22], and frequency stability also improves in real-time PPP time transfer with a receiver clock between-epoch constrained model as described in [23]. In this paper, a simple and well-defined receiver clock model is introduced and tested in the post-processing mode. Furthermore, the constrained PPP is also shown to be able to keep the continuity of the clock solution in case of satellite tracking loss or to retrieve the clock solution from very noisy measurements.

Finally, Atomium integer PPP will be presented. This approach fixes the carrier phase ambiguities as integer numbers in the least square computation. The old version of Atomium PPP in DEMETRA treats the carrier phase ambiguities as float values, ignoring the fact that these ambiguities should be integers. It was reported in [24] that using the carrier phase measurements with integer ambiguity resolution will improve the stability of GNSS time transfer and also eliminate the clock jumps or random walk introduced by float ambiguity solutions. In [25], a new method for fixing integer ambiguity on un-differenced phase measurements was described, and it leads to a PPP with centimeter-level precision. Since the GPS satellite integer products started to be available at the IGS [26], it has become possible for the single receiver user to perform the PPP time transfer with integer ambiguity resolution. Due to the features of the techniques, the independent solution of integer PPP can be only used for frequency transfer, while it cannot be used for time transfer. However, it shows that integer PPP can also perform excellently in long term time transfer, mitigating the problem of time discontinuity at the daily batch boundary if its solution can be calibrated by another time reference [27].

The mathematic details of the three upgrades of Atomium PPP are presented in Section 2, and the associated clock solutions, compared to the current PPP performances, are given in Section 3.

## 2. Updated Atomium PPP Algorithms

### 2.1. Atomium Galileo PPP

As for GPS, the P(Y) code and carrier phase signals ionosphere-free combination on L1 and L2 band are used in the old version of Atomium PPP. The code and carrier phase ionosphere-free measurements of Galileo on E1 and E5a band are added in the new Atomium Galileo PPP computation. The post-processed products of GNSS satellite clocks and orbits are selected for the PPP computation to provide the clock solution with the highest possible stability. To include Galileo, the PPP uses the post-processed satellite products from the IGS in the frame of the MGEX (Multi-GNSS Experiment), which contains products for all the GNSS constellations [28].

To deal with both GPS and Galileo signals in PPP to generate a coherent receiver clock solution, an additional parameter called the inter-system bias (ISB) between GPS and Galileo measurements must be estimated in addition to the antenna position, the receiver clock, and the troposphere delay as the outputs of the least square computation. This bias is the sum of both the differences between the hardware delays in the receiving chain for GPS and Galileo signals and the bias between the time references for the GPS and Galileo satellite clocks in the MGEX products. This ISB is considered as a constant value during the estimation in one daily data batch, based on the assumption that the receiver delay is stable enough during one day, and the same handling scheme is used in the MGEX analysis center [29].

Note that the observed distance between satellite and receiver are measured between the phase centers of the antennas at both ends, while the satellite positions provided by IGS are at the center of mass, they need to be corrected to the phase center positions. The file of IGS phase center corrections for the GNSS antennas at receiver and satellite is utilized. It contains the mean phase center offset and phase center variation corrections and is available on the IGS FTP [30] in ANTEX format (current version is igs14.atx).

The phase center variations applied to GPS and GLONASS satellite antennas are nadir-dependent, while the ones to the Galileo satellite antennas are both azimuth- and nadir-dependent. The azimuth angle of the satellite antenna is estimated in the satellite-fixed coordinate system [31].

The phase center variations for the GNSS receiver antennas are elevation- and azimuth-dependent. However, few antennas are currently calibrated for Galileo signals in the IGS ANTEX file. In this paper, we use the same values in the receiver antenna phase center corrections on GPS L1 and L2 frequencies for the corrections on Galileo E1 and E5a, separately.

Finally, the outputs of Galileo PPP are receiver antenna GPS/Galileo mean phase center position; receiver antenna ARP (antenna reference point) position; receiver clock errors to the MGEX time; troposphere zenith wet delay; ISB.

### 2.2. Atomium Constrained PPP

The Atomium constrained PPP is based on a 2-step process. The first step is a classic PPP. The instantaneous frequency of the PPP clock solution “receiver clock—IGS/MGEX time” is then computed at each epoch as

(1) $$frq_{i}=\frac{clock_{i+1}-clock_{i}}{t_{i+1}-t_{i}},$$

where $clock_{i}$ is the PPP clock solution at epoch $t_{i}$. Then, from these instantaneous frequencies, we estimate the parameters y0 and d of a linear model.

(2) $$frq_{i}=y0+d*t_{i}$$

with y0 the frequency offset and d the frequency drift. The constraint between the clock solutions at adjacent epochs can then be expressed as

(3) $$\begin{array}{l} delt_{i}=clock_{i+1}-clock_{i}\\ \;=\left(y0+d*t_{i}\right)*\left(t_{i+1}-t_{i}\right). \end{array}$$

The estimation variance of the constraint can be predicted as

(4) $$\sigma_{i}{}^{2}=\;{\left(ADev\left(1s\right)\right)}^{2}*\left(t_{i+1}-t_{i}\right),$$

where ADev(1s) is the user input Allan Deviation of the receiver clock at 1 s, which can be found in the clock specification or can be estimated from the clock solution $clock_{i}$. In this paper, we use the ADev(1s) provided in the specification document of the clocks, e.g., 2 × 10^−13^ for a hydrogen maser, 5 × 10^−12^ for a cesium clock. The weight of the clock constraint $delt_{i}$ can be expressed as

(5) $$W_{i}=\;\frac{\sigma^{2}}{\sigma_{i}{}^{2}},$$

where $\sigma^{2}$ is the a posteriori estimation variance on the clock solution at epoch $t_{i}$ from the least square in the classic PPP.

The second step of the approach is then to compute a new PPP, with the additional constraints on the clock solution inserted in the least square computation of PPP. This constraint model is built based on the assumption that the receiver clock is mainly affected by white frequency noise during the analysis period.

In addition, constrained PPP offers the option for outlier recovery. If the recovery mode is on, it assumes that the clock solution is very noisy, the software will remove the frequency outliers before estimating the linear frequency model. With this refined clock constraint, the clock outliers will be pulled back to a normal noise level in PPP computation. If the recovery mode is off, it assumes there are some intentional changes causing a clock jump. In that case, the clock will not be constrained around those jumps, allowing the true clock jump to appear in the clock solution. This recovery mode is on by default, and if it is known there are some physical changes during the day of computation, from user information, it is then switched off.

### 2.3. Atomium Integer PPP

To fix the integer ambiguities of the carrier phase measurements for improved PPP frequency transfer, the GRG satellite products [26] are used in the Atomium integer PPP software instead of the IGS/MGEX products. The GRG products are generated at the CNES (Centre National d’Etudes Spatiales) and CLS (Collecte Localisation Satellites) IGS analysis center. These products include the satellite clock, satellite orbit, earth rotation parameters (ERP), and satellite wide-lane biases (wsb). Since the wsb files for Galileo are not regularly available yet, only GPS signals are included in the current integer PPP software.

The computation takes place in three main steps: 1. Resolve the wide-lane (WL) integer ambiguity; 2. Resolve the narrow-lane (NL) integer ambiguity; 3. Compute the final solution.

1. In the first step, the Melbourne–Wubbena (MW) combination is built at each epoch using phase and code measurements for each GPS satellite:

(6) $$\mathrm{MW}=\left(\alpha_{WL}L1-\beta_{WL}L2\right)-\left(\alpha_{NL}P1+\beta_{NL}P2\right),$$

where (L1, L2) and (P1, P2) represent the carrier phase measurements and code measurements on the L1 and L2 band separately, ($\alpha_{WL}$, $\beta_{WL}$) and ($\alpha_{NL}$, $\beta_{NL}$) are the coefficients in front of the related measurements for the MW combination. Equation (6) can be further expressed as

(7) $$\frac{\mathrm{MW}}{\lambda_{WL}}=\;N_{WL}+WRB-WSB,$$

where $\lambda_{WL}$ is the WL wavelength (in meter), $N_{WL}$ is the integer ambiguity of WL combination (in cycle), WRB is the WL receiver bias, and WSB is the WL satellite bias provided by GRG products.

With the least square method, the real-valued $N_{WL}$ and WRB are estimated by each satellite track and each epoch separately. To avoid the singularity of WRB estimation, an absolute constraint on WRB is set for the first day estimation. Then the GNSS bootstrapping method [32] is adopted to fix the $N_{WL}$ to a closest integer cycle per track, and WRBs are the remaining fractional cycles in the MW combination at each epoch.

In this step, the $N_{WL}$ is fixed only when its success rate of fixing [32] is over 90% and its real-valued ambiguity is not too far from an integer value (smaller than 0.25 WL cycle), only the track with fixed $N_{WL}$ is used for the computation in the next step. To obtain coherent clock solutions for multiple days, the mean of the solved WRB in the previous day is chosen as the constraint on the WRB estimation of the next day. So, the estimated WRBs do not have to be always a fractional cycle of WL as it is evolving, but it has to be continuous to avoid the receiver clock misalignment at different days caused by the integer cycle estimation error of WRB.

2. In step 2, the GPS ionosphere free (IF) code and carrier phase combination can be first expressed as

(8) $$P_{IF}=\;\rho +\;T^{R}+\mathrm{zpd}+e+\;\varepsilon_{P},$$

(9) $$L_{IF}=\rho +\;T^{R}+\mathrm{zpd}+\lambda_{NL}\left(N_{1}+\left(\lambda_{WL}/\lambda_{2}\right)N_{WL}\right)+W+e+\varepsilon_{L},$$

where $P_{IF}$ and $L_{IF}$ are the code and carrier phase ionosphere free combination, respectively, ρ is the distance between satellite and receiver phase center, $T^{R}$ is the receiver clock error, zpd is the troposphere zenith wet delay, W is the wind-up effect to be corrected, e is the other common errors between code and phase measurements (e.g., troposphere dry component delay, relativistic effect, satellite clock error), $\varepsilon_{P}$ and $\varepsilon_{L}$ are the code and phase noises, respectively, $\lambda_{NL}$ is the NL wavelength, $N_{1}$ is the integer ambiguity to be resolved, and integer $N_{WL}$ has already been fixed in step 1.

Since $\lambda_{NL}=17\lambda_{IF}$ = 10.7 cm, it is much easier to resolve the integer ambiguity. The code and carrier phase IF measurements are put in the least square to compute the real-valued $N_{1}$ per track, and the integer $N_{1}$ is fixed again by the GNSS bootstrapping method. The $N_{1}$ ambiguities are also partially fixed as in step 1, the measurements with un-solved ambiguities are excluded from the next computation.

3. In the last step, since the integer ambiguities of the carrier phase IF combination have already been resolved, the carrier phase IF measurement is not ambiguous anymore and is rewritten again as

(10) $$L_{IF}-\lambda_{NL}\left(N_{1}+\left(\frac{\lambda_{WL}}{\lambda_{2}}\right)N_{WL}\right)-W-e=\rho +T^{R}+\mathrm{zpd}+\varepsilon_{L},$$

where the variables on the left side are already solved, the ones on the right side are to be solved, including receiver position (in ρ), receiver clock error, and troposphere error. The wind-up corrections are computed from step 2, together with the ambiguity fixing.

At last, the carrier phase IF measurements are put in the least square without code IF measurements to solve the final solutions.

If the WRB estimation is coherent during the whole period and the ambiguities are resolved correctly, the values of the discontinuities at the daily boundaries for the integer PPP clock solutions are the integer multiple of the GPS $\lambda_{NL}$. Hence, the clock solutions for multiple days can be easily aligned with minimal error introduced. Attention also needs to be paid to the discontinuity inside the daily batch. If it is caused by any a hardware change in the receiver system, the integer feature of discontinuity is lost because the WRB may endure an integer cycle estimation error in Equation (7) after the discontinuity, and bias the solution in Equation (10) in terms of an integer multiple of $\lambda_{NL}\left(\lambda_{WL}/\lambda_{2}\right)N_{WL}$.

## 3. Processing and Results

In this section, the performances of the updated PPP software are evaluated in several cases of remote clock comparison. To focus on the performance of the software themselves without the effect of the quality of the compared clocks, two kinds of experiments were built: 1. Common clock difference (CCD), where the two GNSS stations are driven by the same external clock; 2. “PPP–OPT”, which means the difference of clock comparison results from PPP and optical fiber link.

The purpose of both experiments was to cancel the effect of the clocks themselves. The CCD results were assumed to be around zero if both GNSS stations were perfectly calibrated since it is the common clock that was compared. Two CCD experiments were set: 1-month common clock comparison using stations GR01 and GR02 in INRIM during the modified Julian date (MJD) 58591–58636 and another comparison using stations BRUX and RTBS in ORB during MJD 58583–58621. “PPP–OPT” removes the clock component by subtracting the clock comparison results from two individual techniques, in which the optical fiber link results are expected to be more stable and accurate than the GNSS results. The astrogeodynamic observatory of the space research center (AOS) and the central office of measures (GUM) provide a continuous optical link comparison between their atomic clocks [33]. Meanwhile, the clocks are also compared through their GNSS stations AO_4 and GUM4 using NRCAN PPP (both measurements can be downloaded from the BIPM FTP ftp://tai.bipm.org/TimeLink/LKC/ [6]). Two periods of GNSS observation data and available measurements from both the optical link and NRCAN PPP at AOS and GUM were collected for the “PPP–OPT” experiment: MJD 58417–58447 and MJD 58537–58572. For the stations in the CCD experiments, the satellite MGEX and GRG products from CNES/CLS IGS analysis center were used, and for station AO_4 and GUM4, which only receive GPS signals, the IGS rapid products were used for the study of constrained PPP, and the GRG products from CNES/CLS were used for the integer PPP clock comparison.

Note that the accuracy of the GNSS station calibration is not within the scope of this paper, only the stability and precision of the clock solution are studied here. Actually, these calibration values are normally constant values during clock comparison, which introduces only an offset to the GNSS clock output from the software but does not affect the stability. In this section, all the clock comparison results were shifted towards zero to ease the comparison between different solutions.

### 3.1. Atomium Galileo PPP

**Figure 1:**
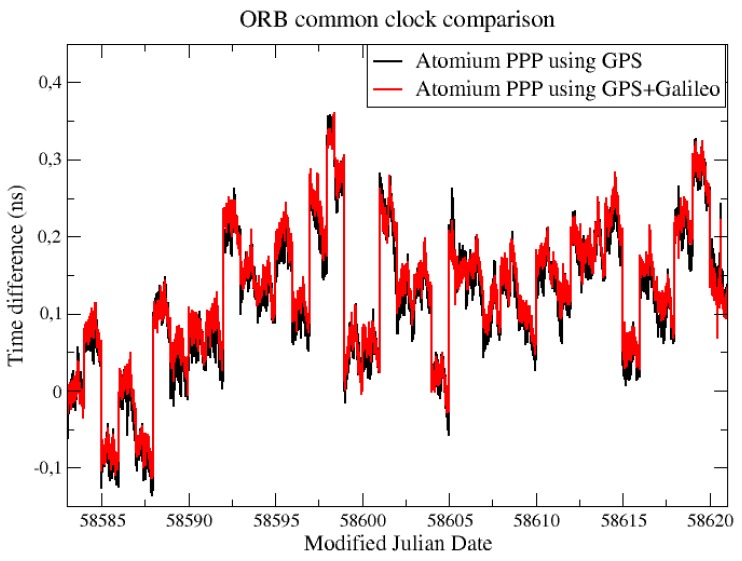
Common clock difference (CCD) Galileo results between BRUX and RTBS.

For both the CCD experiments in ORB and INRIM, the common clock was compared through their two GNSS stations with the Atomium PPP using GPS signals and GPS + Galileo signals. As shown in Figure 1, the two solutions in ORB show a good coherence, however, due to the presence of daily boundary jump, the performances of the GPS-only solution and GPS + Galileo solution are hard to compare.

**Figure 2:**
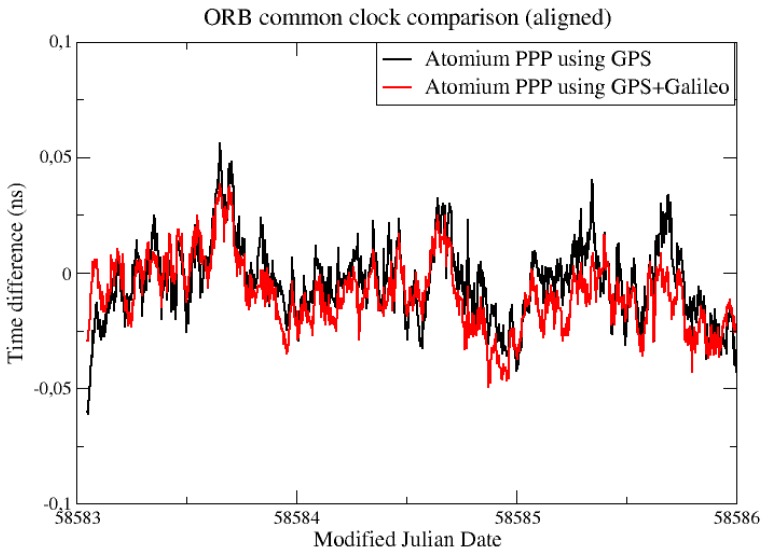
3 days aligned CCD Galileo results from Figure 1.

For a better comparison, the daily batch boundaries were minimized by aligning the daily results using 2nd order extrapolation at the border [6]. This method will cause an accumulated error in the long-term, so only 3 days of data from Figure 1 were aligned and compared, as displayed in Figure 2.

**Figure 3:**
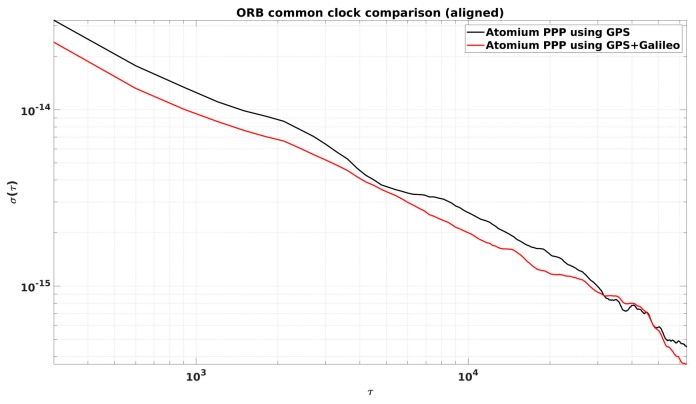
Allan DEViations (ADEVs) for the CCD global positioning system (GPS)-only and GPS + Galileo precise point positioning (PPP) results in the Observatory of Belgium (ORB) from Figure 2.

**Figure 4:**
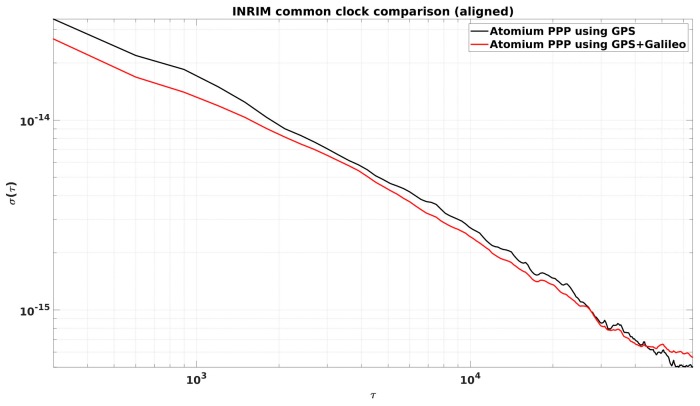
ADEVs for the CCD GPS-only and GPS + Galileo PPP results in the Italian National Metrology Institute (INRIM).

From Figure 2, it is observed that the daily PPP clock comparison noise was generally within 0.1 ns, and with GPS + Galileo signals, the PPP obtained slightly more precise results, and a similar conclusion can be made from the CCD experiments in INRIM. To better elaborate the improvement by adding Galileo signals, they were compared in the form of Allan DEViation (ADEV). The ADEVs for the CCD results in ORB and INRIM are plotted in Figure 3 and Figure 4.

The ADEVs of GPS + Galileo PPP results were generally lower than the GPS-only ones, as shown in Figure 3 and Figure 4. The improvement in the frequency stability at medium and short averaging times was generally between 10% and 25%. However, due to the discontinuity at the batch boundary, as shown in Figure 1, it is hard to compare the long-term stability.

### 3.2. Atomium Constrained PPP

**Figure 5:**
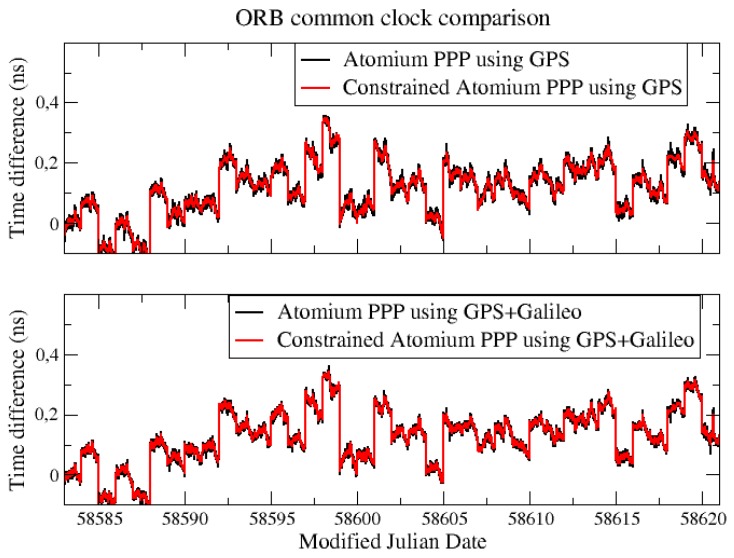
CCD constrained results in ORB.

**Figure 6:**
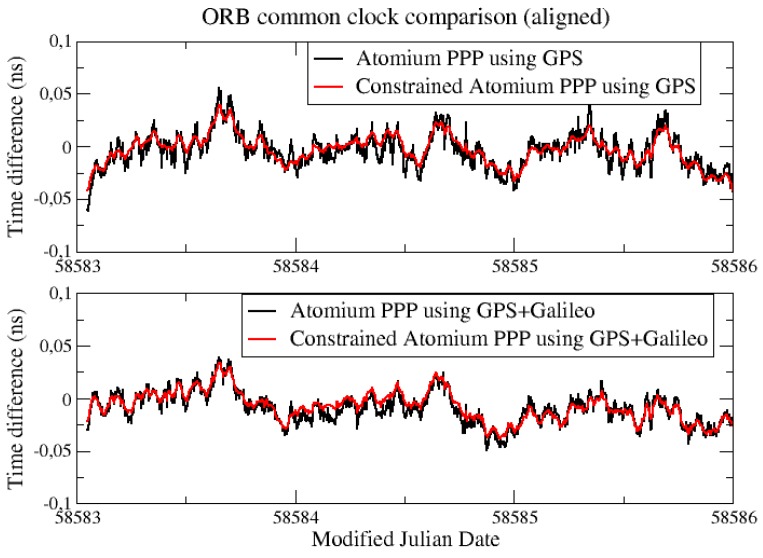
Aligned CCD constrained results in ORB.

The constraints were applied to GPS-only PPP and GPS + Galileo PPP results separately, and the results were compared with the original clock results in Figure 5. Both INRIM and ORB have their hydrogen maser clock for the comparison in this experiment, and the values of ADev(1s) were both set to 2 × 10^−13^. As seen in Figure 5, the constrained PPP can reduce the noise of the clock solution inside the batch and keep the long-term stability as for the original PPP. The noise reduction for the constrained PPP was more visible in an aligned solution using 3 days’ clock data, as shown in Figure 6.

**Figure 7:**
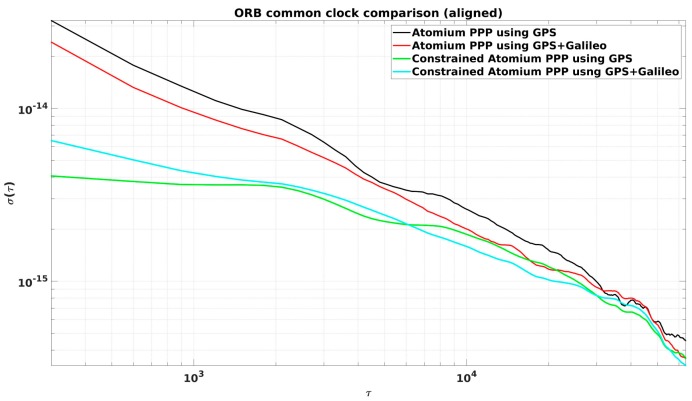
ADEVs of aligned CCD results in ORB.

**Figure 8:**
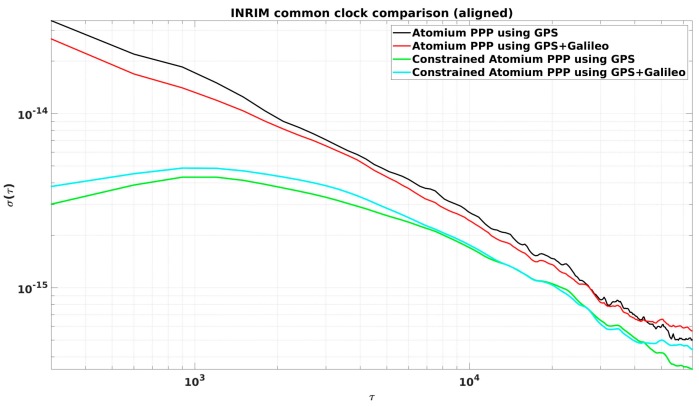
ADEVs of aligned CCD results in INRIM.

The ADEVs of constrained PPP, using the aligned 3-day results, were compared with the classical PPP ones in Figure 7 and Figure 8 for both the CCD experiments in ORB and INRIM. 

It is very clear that constrained PPP improves clock frequency stability (see Figure 7 and Figure 8), especially at short averaging times, as reported in Table 1. It can be observed in both Figures that the best short-term stability was provided by GPS-only constrained PPP, rather than the GPS + Galileo constrained PPP. It is most probably because the GPS + Galileo PPP has more satellite measurements at each epoch, and the relative weight of the constraint gets smaller for GPS+Galileo than for GPS-only.

**Figure 9:**
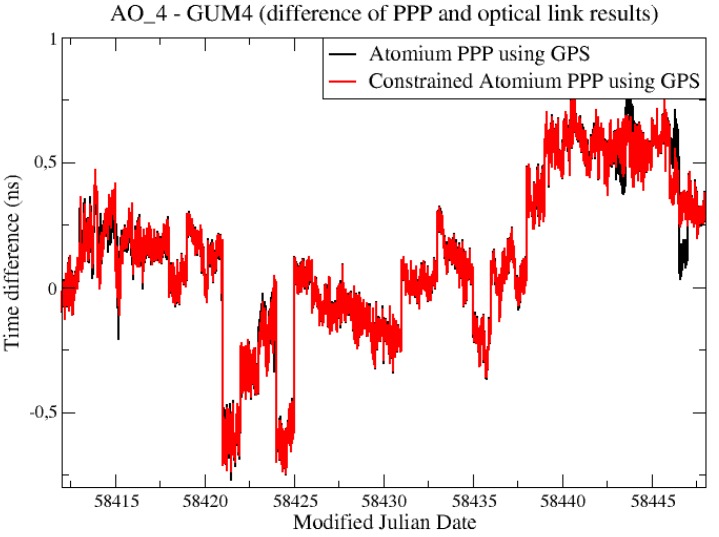
“PPP—OPT” experiments to evaluate constrained PPP.

The “PPP—OPT” experiments were also built here to test the constrained PPP. Since both AO_4 and GUM4 stations receive only GPS signals, GPS + Galileo PPP results were not available for the following “PPP—OPT” experiments. The GPS-only results were compared in Figure 9.

**Figure 10:**
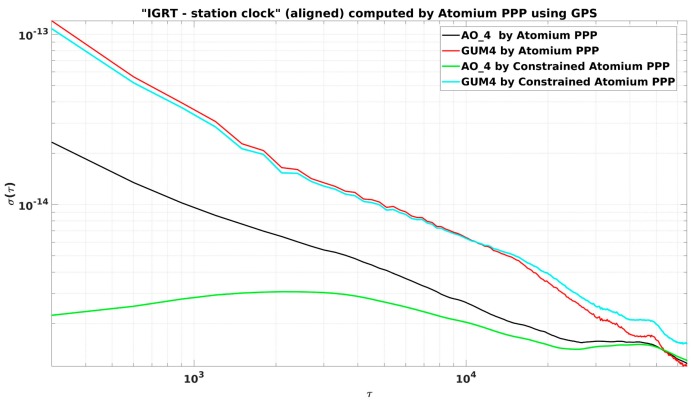
ADEVs in “PPP—OPT” experiments to evaluate constrained PPP. IGRT stands for the reference time of International global navigation satellite systems service (IGS) rapid products that are used in the PPP computation.

It was found (Figure 9) that the constrained PPP did not reduce the noise obviously as in the former CCD experiments. The reason is that, while the AO_4 is driven by a hydrogen maser, GUM4 is driven by a caesium clock. As a result of its lower ADev(1s), the caesium clock will be constrained in PPP with a much smaller weight than a hydrogen maser, as we can see from Equations (4) and (5). Although the constraint improved the stability dramatically at the AO_4 side, the caesium clock estimation at the GUM4 side was not improved effectively by the constraint, as shown in Figure 10. The noise of caesium clock dominated the stability of the two clock comparison so that the final constrained PPP solution did not show any improvement.

In addition to the normal function of constrained PPP, the applications of constrained PPP in different scenarios were also studied in this paper:

#### 3.2.1. The Signal Tracking Loss

**Figure 11:**
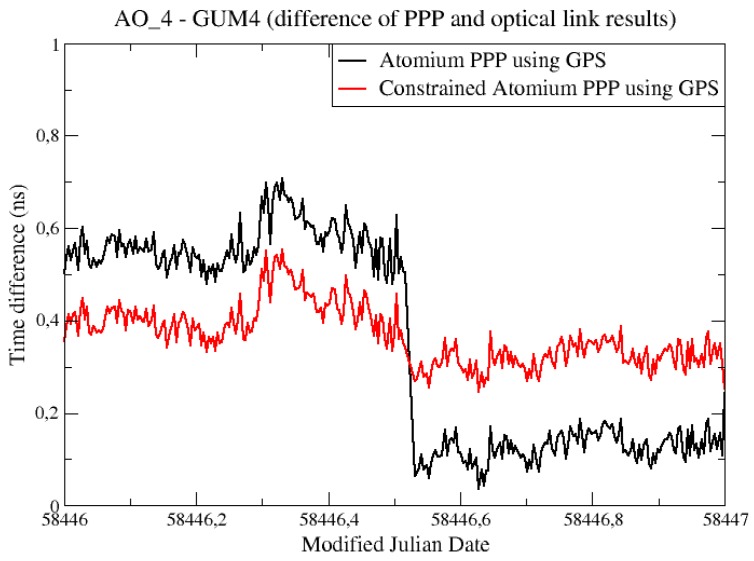
“PPP—OPT” experiments to evaluate constrained PPP.

There was a notable difference between the constrained PPP and non-constrained PPP results in the last test periods of the former “PPP—OPT” experiment, as can be seen in Figure 9. These differences were zoomed in and plotted again in Figure 11.

The jump that happens in the middle of the MJD 58446 was not a daily batch jump, but because all the satellite tracks restarted so that new ambiguities were determined for all the satellites, at that epoch in the PPP computation for station AO_4, and the jump happened just as at the batch boundary. This jump was overcome by the constrained PPP, in which the receiver clock measurements were relatively constrained at all epochs.

#### 3.2.2. For an Extreme Noisy Environment 

**Figure 12:**
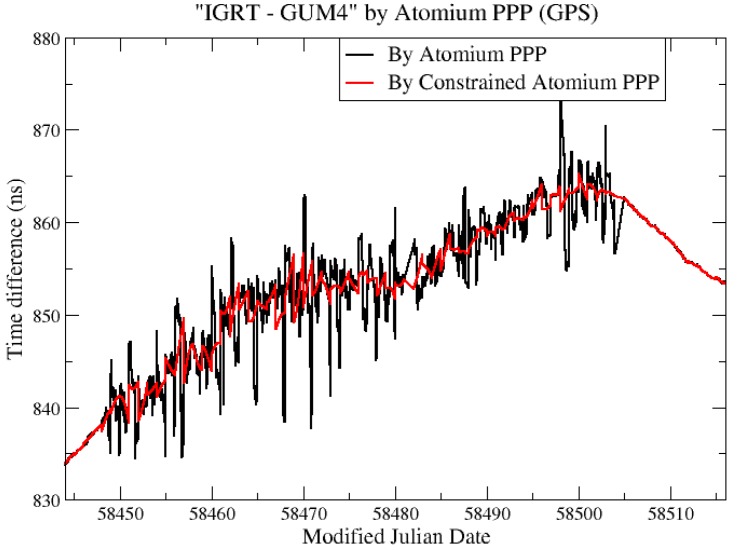
“IGRT—GUM4” from constrained and non-constrained PPP.

Station GUM4 was observed to have very noisy receiver measurements for around 2 months, and it returned to normal after some receiver modification. The basic idea was constraining the measurements’ noise by constrained PPP, as illustrated in Figure 12.

Since the cesium clock estimation at GUM4 was not affected much by the constraint model as mentioned before, a larger weight of the clock constraint at GUM4 was set in this experiment to further restrict the abnormal measurements’ noise (the same weight as for the hydrogen maser was chosen). The constrained PPP reduced the noise but still not at a satisfactory level, which can be seen in Figure 12. This is because the frequency offset and drift over each day were estimated with larger uncertainties due to the very noisy measurements.

To further retrieve the correct clock solution from the noisy environment, we needed a more accurate frequency measurement of the clock. For this purpose, we estimated the averaged frequency deviation “IGRT—GUM4” (Frq_“IGRT—GUM4”_) as the difference of Frq_“IGRT—AO_4”_ and Frq_“AO_4—GUM4”_. Frq_“IGRT—AO_4”_ was computed from the clock solution IGRT—AO_4 through Atomium PPP, and Frq_“AO_4—GUM4”_ was computed from independent clock comparison results of AO_4—GUM4. Then the constraints were computed from the frequency data and applied to Atomium PPP to estimate the constrained “IGRT—GUM4”. In the present case, we used the optical link for AO_4—GUM4. This link is even more stable than the PPP, so there is no real advantage of using it to get a constrained PPP solution. However, in some other links, the only available independent link might be a two-Way satellite time and frequency transfer (TWSTFT) [34], which provides only 24 data points per day, and therefore, does not provide short-term stability. In that case, using the TWSTFT to get the frequency deviation of the link will be very helpful to get a constrained PPP, and hence, a frequency transfer with very good short-term frequency stability.

**Figure 13:**
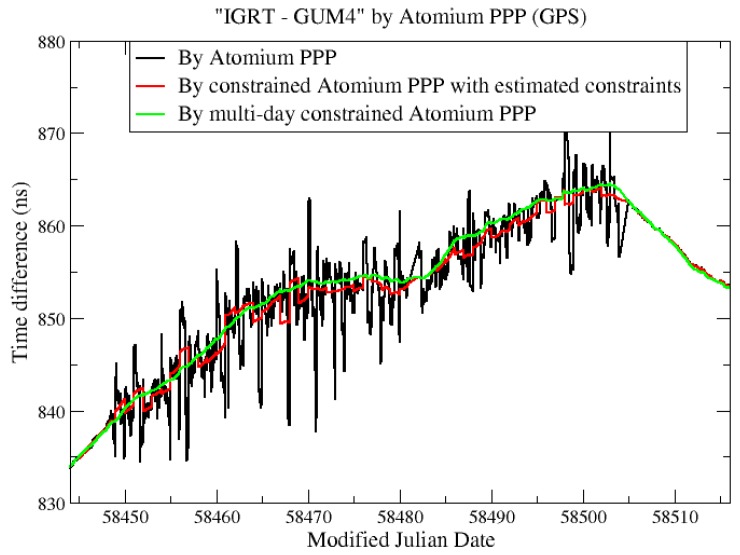
“IGRT—GUM4” from PPP and constrained PPP.

The results of constrained PPP at GUM4 using estimated constraints are displayed in the red line in Figure 13. To further mitigate the daily boundary effect, and also to verify the correctness of the constrained clock results inside the batch, the multi-day constrained PPP computations were also performed: For each daily batch, in addition to the relative constraints, the receiver clock at the first epoch was further absolutely constrained based on the estimated clock at the last epoch of the previous batch and the increment predicted from the frequency model of the previous batch. The corresponding multi-day constrained PPP results are plotted in Figure 13 in the green line. It is observed from Figure 13 that the constrained PPP using the estimated constraints can highly reduce the clock measurements’ noise, and the multi-day constrained PPP can further provide continuous clock solutions by reducing the size of the daily boundary jump. However, the multi-day constrained method should be used carefully since it may also introduce an artificial variation up to hundreds of ps during 1-month clock measurements. Hence, it is recommended to use this multi-day constrained PPP in extremely noisy environments only to restrict the large daily boundary jump, which is the main factor influencing the stability of the constrained PPP clock solution, as can be seen in Figure 13.

The correct frequency data are expected to be obtained from external products, e.g., TWSTFT. Though it is also possible to directly use the phase data from external products if it exits, the calibration error of the external measurements will also be introduced. Meanwhile, the estimated frequency data is not affected by these calibration errors and can be used by the target clock without any bias.

### 3.3. Atomium Integer PPP

**Figure 14:**
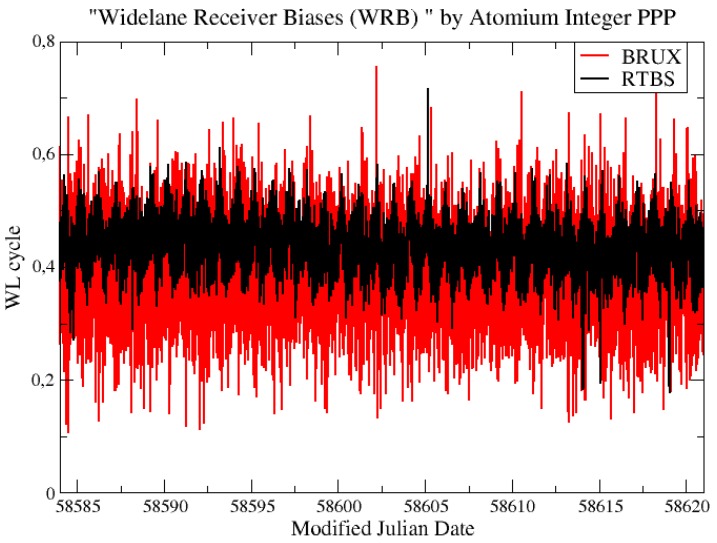
Wide-lane receiver bias (WRB) at station BRUX and RTBS estimated by integer PPP.

As described before, to check if there was any integer cycle estimation error of $N_{WL}$ in the integer PPP results, the continuity of the estimated WRB needs to be ensured. Figure 14 shows the estimated WRB at the first step of integer PPP for the station BRUX and RTBS in the CCD experiments, and no integer cycle error was found during the period of estimation.

**Figure 15:**
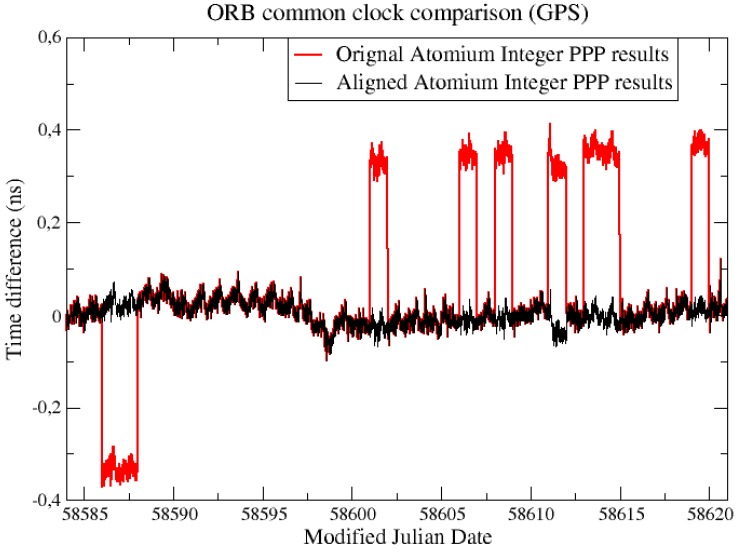
CCD experiments at ORB with integer PPP.

As shown in Figure 15, the common clock comparison results using integer PPP also showed some discontinuities at the daily batch boundaries. However, these discontinuities were only integer multiples of the GPS $\lambda_{NL}$ (are 1 or −1 $\lambda_{NL}$ in Figure 15), and the continuous clock solution could be easily obtained after alignment by adding integer multiples of $\lambda_{NL}$.

**Figure 16:**
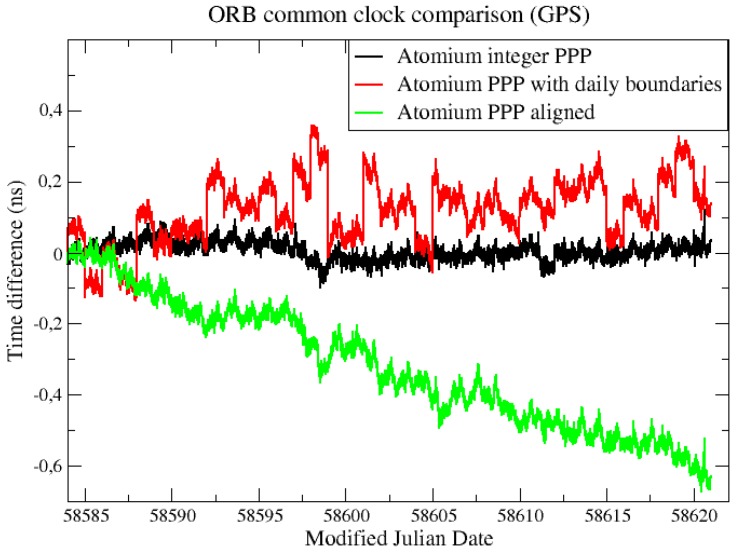
CCD results in ORB using Atomium integer PPP and original Atomium PPP.

**Figure 17:**
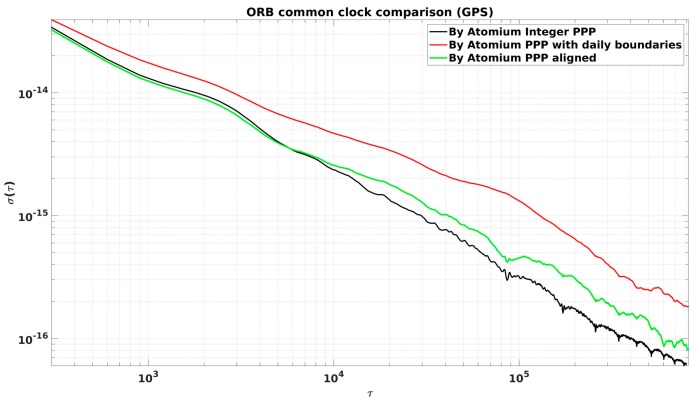
ADEVs between integer PPP and PPP CCD results in ORB.

This continuous integer PPP CCD result was compared to the original Atomium PPP result with daily boundary jumps and the aligned original Atomium PPP result in terms of phase offset and ADEVs, as illustrated in Figure 16 and Figure 17, separately. Compared to the original Atomium PPP results, integer PPP clearly improved the frequency stability from several hours to longer averaging times, and the frequency accuracy of $1\;\times \;10^{-16}$ could be reached by 4 days averaging. Aligning the daily Atomium PPP results using 2nd order extrapolation to remove the daily boundary jumps could largely improve the frequency stability as shown in Figure 17, however it caused a drift in the time solution, as shown in Figure 16 (where the aligned data in green line accumulate an error of about 0.6 ns in a month). It is, therefore, not recommended to align the daily time solutions from float ambiguities PPP over a long time span to not affect the time accuracy.

The improvements of frequency stability at different averaging times by using Atomium integer PPP instead of Atomium PPP (corresponding to the black and red lines in Figure 17) are recorded in Table 2. It is shown that the largest improvement happened at the averaging time of one day, which was exactly the occurrence rate of the daily boundary jump.

**Figure 18:**
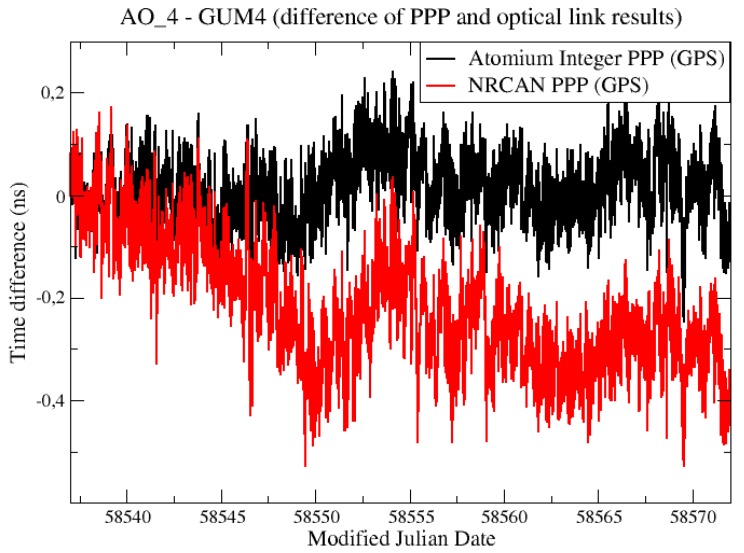
Comparison of integer PPP and NRCAN PPP results.

Figure 18 compares the “PPP—OPT” results from integer PPP with the one from NRCAN PPP, which was based on a multi-day batch to avoid the daily jumps within the batch. The NRCAN PPP results showed a slight divergence and reach 0.4 ns at the end of the one-month batch.

**Figure 19:**
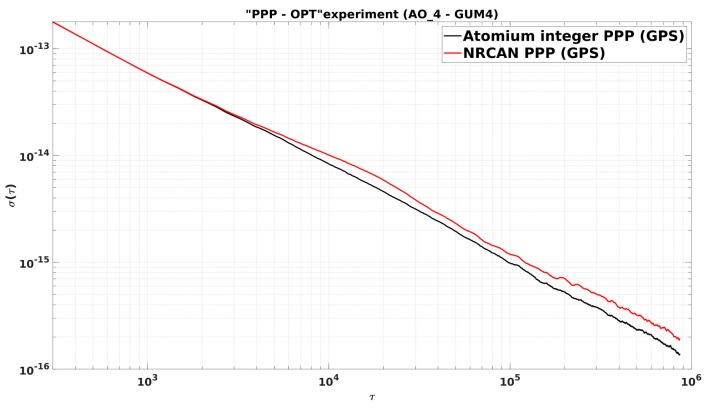
ADEVs between integer PPP and NRCAN PPP results.

The ADEVs of the clock measurements in Figure 18 are further compared in Figure 19. A similar conclusion can be made as from the CCD experiments: The integer PPP improved the clock frequency stability from an average of 2.5 h to longer times compared to the NRCAN PPP, and at 10 days averaging, the stability of $1\;\times \;10^{-16}$ was acquired by using integer PPP.

## 4. Conclusions

Three updated versions of Atomium PPP software were presented in this paper: Atomium PPP with GPS + Galileo measurements; Atomium PPP with receiver clock constrained model; Atomium PPP with integer ambiguity resolution. Two kinds of experiments were built to test the performance of their clock solutions. Using Galileo in addition to GPS in PPP computation reduced the noise of the clock solution inside the daily batches, while the long-term stability was hardly improved due to the presence of daily jumps. The basic function of constrained PPP is to reduce the short-term noise of the clock measurements on good quality clocks as hydrogen masers, by restraining the short-term thermal noise. In addition, two more application scenarios for constrained PPP were introduced in this paper. First, constrained PPP can keep providing continuous clock solution during the period when the receiver experiences short-term loss of lock. Second, constrained PPP can be used to retrieve true clock solutions from very noisy measurements as long as the related clock frequency data can be estimated from external products, e.g., from TWSTFT or optical link measurements, without introducing their calibration errors at the phase measurements. Finally, integer PPP shows an advantage to the current float ambiguity PPP techniques on the clock frequency transfer stabilities at mid-term to long-term averaging times; a frequency stability of $1\;\times \;10^{-16}$ can be reached at an averaging of 4 days to 10 days, depending on the time link. It is expected that when the Galileo integer ambiguity satellite products are regularly available, the integer PPP with GPS + Galileo will further improve the current GNSS clock frequency transfer capacity.

Currently, all the updated Atomium PPP software have been integrated into the DEMETRA server to provide the daily solutions for users who need post-processing services with better performances. GPS and Galileo daily quality monitoring data are both available for the users. And the test and the integration of the Atomium Galileo PPP hourly solution is in progress.

## Figures and Tables

**Table 1:** The stability improvements when applying constraint model to global positioning system (GPS) precise point positioning (PPP) (black to the green line in Figure 7 and Figure 8) and GPS + Galileo PPP (red to the light blue line in Figure 7 and Figure 8), respectively.

Averaging Time	Improvement of Stability	
	GPS	GPS + Galileo
5 min	89.3%	79.4%
1 h	50.0%	36.7%
3 h	32.3%	23.1%
12 h	21.1%	17.7%

**Table 2:** The stability improvements applying integer ambiguity resolution to Atomium PPP (GPS).

Averaging Time	Improvement of Stability
5 min	13.3%
1 h	31.2%
3 h	50.2%
12 h	63.3%
1 day	80%
5 days	65.3%
